# DNA hydroxymethylation is associated with disease severity and persists at enhancers of oncogenic regions in multiple myeloma

**DOI:** 10.1186/s13148-020-00953-y

**Published:** 2020-11-02

**Authors:** Jean-Baptiste Alberge, Florence Magrangeas, Mirko Wagner, Soline Denié, Catherine Guérin-Charbonnel, Loïc Campion, Michel Attal, Hervé Avet-Loiseau, Thomas Carell, Philippe Moreau, Stéphane Minvielle, Aurélien A. Sérandour

**Affiliations:** 1 Université de Nantes, CNRS, Inserm, CRCINA, Nantes, France; 2 Site de Recherche Intégrée Sur Le Cancer (SIRIC) ILIAD, INCA-DGOS-Inserm 12558, Nantes, France; 3 Hematology Department, University Hospital, Nantes, France; 4 Ludwig Maximilian Universität München, Munich, Germany; 5 Institut de cancérologie de L’Ouest Site René-Gauducheau, Saint-Herblain, France; 6 Centre de Recherche en Cancérologie de Toulouse, Institut National de la Santé et de la Recherche Médicale U1037, Toulouse, France; 7 Unit for Genomics in Myeloma, Institut Universitaire du Cancer de Toulouse-Oncopole, University Hospital, Toulouse, France; 8 École Centrale de Nantes, Nantes, France

**Keywords:** Multiple myeloma, Epigenetics, DNA modifications, Hydroxymethylation

## Abstract

**Background:**

Multiple myeloma (MM) is a heterogeneous plasma cell malignancy that remains challenging to cure. Global hypomethylation correlates with an aggressive phenotype of the disease, while hypermethylation is observed at particular regions of myeloma such as B cell-specific enhancers. The recently discovered active epigenetic mark 5-hydroxymethylCytosine (5hmC) may also play a role in tumor biology; however, little is known about its level and distribution in myeloma. In this study, we investigated the global level and the genomic localization of 5hmC in myeloma cells from 40 newly diagnosed patients, including paired relapses, and of control individuals.

**Results:**

Compared to normal plasma cells, we found global 5hmC levels to be lower in myeloma (*P* < 0.001). Higher levels of 5hmC were found in lower grades of the International Staging System prognostic index (*P* < 0.05) and tend to associate with a longer overall survival (*P* < 0.1). From the hydroxymethylome data, we observed that the remaining 5hmC is organized in large domains overlapping with active chromatin marks and chromatin opening. We discovered that 5hmC strongly persists at key oncogenic genes such as *CCND1*, *CCND2* and *MMSET* and characterized domains that are specifically hydroxymethylated in myeloma subgroups. Novel 5hmC-enriched domains were found at putative enhancers of *CCND2* and *MYC* in newly diagnosed patients.

**Conclusions:**

5hmC level is associated with clinical aspects of MM. Mapping 5hmC at a genome-wide level provides insights into the disease biology directly from genomic DNA, which makes it a potent mark to study epigenetics on large patient cohorts.

## Introduction

Multiple myeloma (MM) is a plasma cell (PC) neoplasm with an incidence rate of 5/100,000 in Europe and it accounts for approximately 1% of all cancers. Median survival of patients has greatly improved in the last decade [1] with the use of novel strategies such as autologous stem cell transplantation and new sets of drugs: immunomodulators, proteasome inhibitors, histone deacetylase (HDAC) inhibitors and monoclonal antibodies [2]. Yet, the treatment remains challenging as nearly all patients ultimately relapse with the emergence of a resistant subpopulation of malignant plasma cells. Malignant clones show a heterogeneous range of mutations and chromosomal abnormalities along with heterogeneous chromatin and epigenetic dysregulations at diagnosis and relapse that affects biological pathways such as MAPK, NF-κB, or DNA-repair [3].

Genomic and transcriptomic studies have allowed a better understanding of the disease and identified key transcription factors (TFs) involved, such as IRF4, MYC, PRDM1 and XBP1 [3]. Recent epigenomics technologies can help to deepen our knowledge of the transcriptional programs shaping MM. However, epigenomics analysis through histone marks profiling with chromatin immunoprecipitation (ChIP-seq) or open chromatin mapping with assay for transposase-accessible chromatin (ATAC-seq) can be hard to set up on a cohort with limited material over a long-time frame and thus gives a limited insight into the disease’s establishment and relapse. Studying DNA epigenetics marks is more adapted to this challenge.

Oxidative states of 5-methylated Cytosine (5mC) such as 5hmC, 5fC and 5caC were identified in genomic DNA a decade ago [4–6]. While DNMT1/3A/3B catalyzes DNA methylation at CpG, the TET proteins TET1/2/3 are responsible for the 5mC oxidation. Interestingly, TET1 and TET2 have been shown to have tumor suppressor roles in B cell lymphomas [7, 8]. 5fC and 5caC are almost undetectable in genomic DNA unless the glycosylase TDG gene is knocked-out [9, 10], whereas 5hmC can be found in all cell types at various levels [11]. 5hmC is believed to be a DNA demethylation intermediate in a process involving TET proteins, TDG and the Base Excision Repair system [12]. However, 5hmC has also shown to be in most cases a stable DNA modification and its abundance increases with DNA age [13, 14]. 5hmC is commonly accepted as a DNA mark associated with active chromatin [15–17] and is a powerful way to identify active genomic domains associated with a disease directly from genomic DNA or more recently from circulating DNA [18, 19].

In solid tumors, 5hmC is depleted compared to normal tissue [20] and some studies show that a lower 5hmC level is associated with poorer outcome [21–23]. When 5hmC is depleted, the mark is lost at genic regions, particularly around transcription start sites (TSS) and in gene bodies [24]. However, the putative tumor suppressor role of 5hmC is poorly characterized [20, 25].

Agirre et al. [26] described DNA methylation in an important number of MM samples. They identified very heterogeneous levels of methylation from one patient to another. They found that despite a global hypomethylation [27–29], local and extensive hypermethylation is present in MM at intronic enhancer regions that are associated with B cell-specific enhancers and stem cell development.

Chatonnet et al. [30] recently identified several hydroxymethylated CpGs in a limited panel of MM samples, yet the genome-wide mapping of 5hmC (hydroxymethylome) has never been studied in a well-established cohort.

In this study, we quantified the 5mC and 5hmC by MS and mapped the 5hmC genome-wide on plasma cell DNA from 40 patients newly diagnosed with MM between 2010 and 2012 [31] and representative of the main molecular sub-types, including 4 paired relapse samples, and of the plasma cells of 5 control individuals.

## Results

### 5hmC negatively correlates with disease severity in newly diagnosed multiple myeloma

We studied a cohort of 40 patients newly diagnosed with multiple myeloma (NDMM) between 2010 and 2012 and 5 healthy bone marrow donors. NDMM were included in the “Intergroupe Francophone du Myélome/Dana Farber Cancer Institute 2009” cohort (IFM/DFCI) and have expression profile available [32]. Patients’ characteristics are described in Additional file 1: Table S1.

**Fig. 1:**
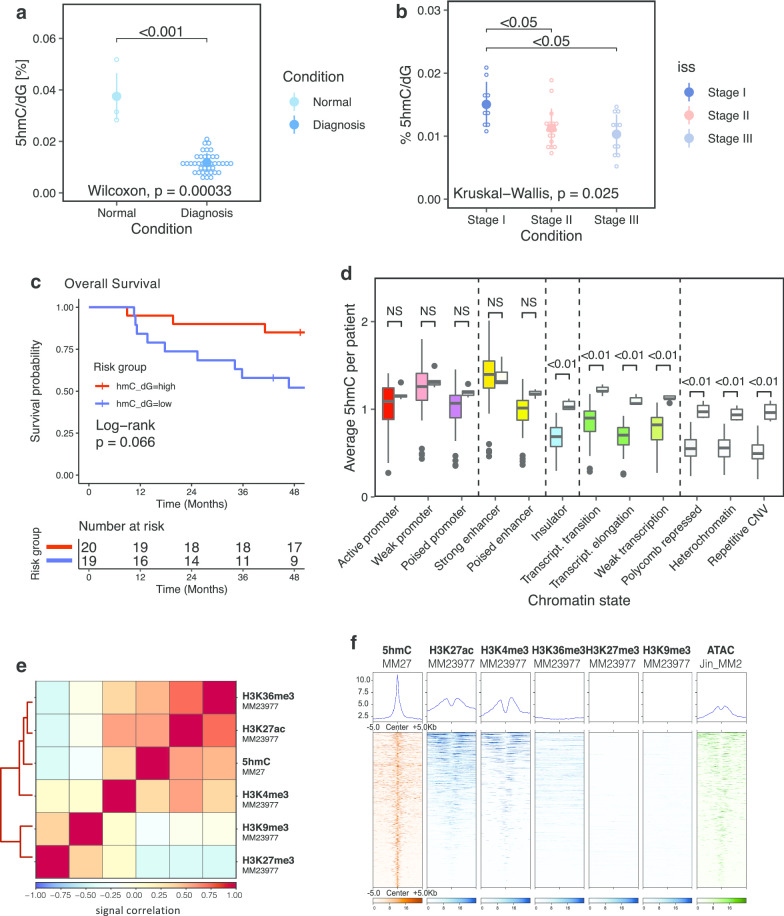
DNA hydroxymethylation is depleted in NDMM and remains locally at active chromatin. **a** Dot plot of 5hmC global quantification by MS in normal plasma cells from healthy donors (*n* = 5), and of myeloma cells of patients at diagnosis (*n* = 39) and **b** at disease stage (ISS I *n* = 9; ISS II *n* = 17; ISS III *n* = 12; NA *n* = 1). **c** 5hmC level-based survival analysis with separation of two groups of NDMM (*n* = 20 and 19, 1 measurement failure) **d** Distribution of the 5hmC signal at the different ChromHMM chromatin states in multiple myeloma (*n* = 40, each left-side boxplot) versus normal plasma cells (*n* = 5, right sides). **e** Correlation matrix between 5hmC signal (this study) and the histone marks H3K36me3, H3K27ac, H3K4me3, H3K9me3 and H3K27me3 (ChIP-seq data from the Blueprint Consortium) in MM patients. **f** Average signal and signal matrix at 5hmC peaks of 5hmC (this study), histone marks (Blueprint) and ATAC-seq [33] in MM patients. ISS, International Staging System, NDMM, Newly Diagnosed Multiple Myeloma

We quantified by MS the global level of 5mC and 5hmC in 49 samples (40 diagnosis in 5mC, 39 in 5hmC with one failure, 4 relapses and 5 normal plasma cell samples). We found that both 5mC and 5hmC were significantly reduced in MM compared to normal plasma cells (NPC) (Fig. 1a and Additional file 2: Figure S2A). On average, 5mC is reduced (*P* < 0.001) by 34%, while 5hmC is reduced (*P* < 0.001) by 69% in MM with respect to NPC.

We found that 5hmC, but not 5mC, is reduced in MM stages II and III compared to Stage I (resp. by 25% and 31%, *P* < 0.05) regarding the International Staging System [34], a classification of patients based on beta2-microglobulin and albumin levels with a strong prognosis value (Fig. 1b right and Additional file 2: Figure S2B). 5mC and 5hmC global levels were not correlated to the sex or the age of the patients (Additional file 2: Figure S2C–F).

Survival analysis was performed on the global level of 5mC and 5hmC measured by MS. Higher 5hmC global level tends to be associated with good outcome (hazard ratio (HR) = 2.6, CI = [0.9, 7.8], *P* = 0.066) (Fig. 1c), whereas 5mC global level does not show significant association with survival (*P* = 0.26, Additional file 2: Figure S3).

### 5hmC persists at active and accessible chromatin of myeloma cells

These results encouraged us to characterize the hydroxymethylome by 5hmC-seq [35] in the 40 NDMM and 5 NPC to identify the genomic regions remaining marked by 5hmC.

The 5hmC signal predominantly marks strong enhancers, promoters, and to a lesser extend transcribed regions of NDMM (Fig. 1d, each left-side boxplot) when we used a ChromHMM functional annotation based on modified histone ChIP-seq data from the NCI-H929 cell line. Insulator, Polycomb repressed, heterochromatin and repetitive regions are depleted in 5hmC in NDMM compared with the strong enhancers and promoters. In NPC however, 5hmC is relatively widespread (Fig. 1d, each right-side boxplot). When comparing NDMM and NPC, we show that 5hmC is globally depleted in the genome but persists in active promoters and enhancers (Fig. 1d).

Furthermore, we found 5hmC to be positively correlated with active chromatin (H3K27ac and H3K36me3, in a less extend with H3K4me3) but negatively correlated with inactive chromatin (H3K9me3 and H3K27me3) in independent MM patients (Fig. 1e). At local enriched regions, we found the 5hmC peaks to be associated with H3K27ac, H3K4me3 and broad H3K36me3 signals but not with H3K9me3 and H3K27me3 (Fig. 1f). The 5hmC signal is located in H3K27ac and H3K4me3 valleys proximal to histone mark peaks.

The 5hmC peaks are also associated with open chromatin as it is enriched in ATAC-seq signal (Fig. 1f). Finally, we observed that the level of 5hmC signal is enriched within gene bodies of highly transcribed genes, as expected from previous studies [16, 35, 36] (Additional file 2: Figure S4A).

All together, these results show that despite its global loss in NDMM compared to NPC, 5hmC is present at transcriptionally active chromatin. This makes 5hmC a useful tool to study active chromatin in DNA from patients’ samples.

### The 5hmC landscape in MM is organized in large 5hmC-enriched domains

**Fig. 2:**
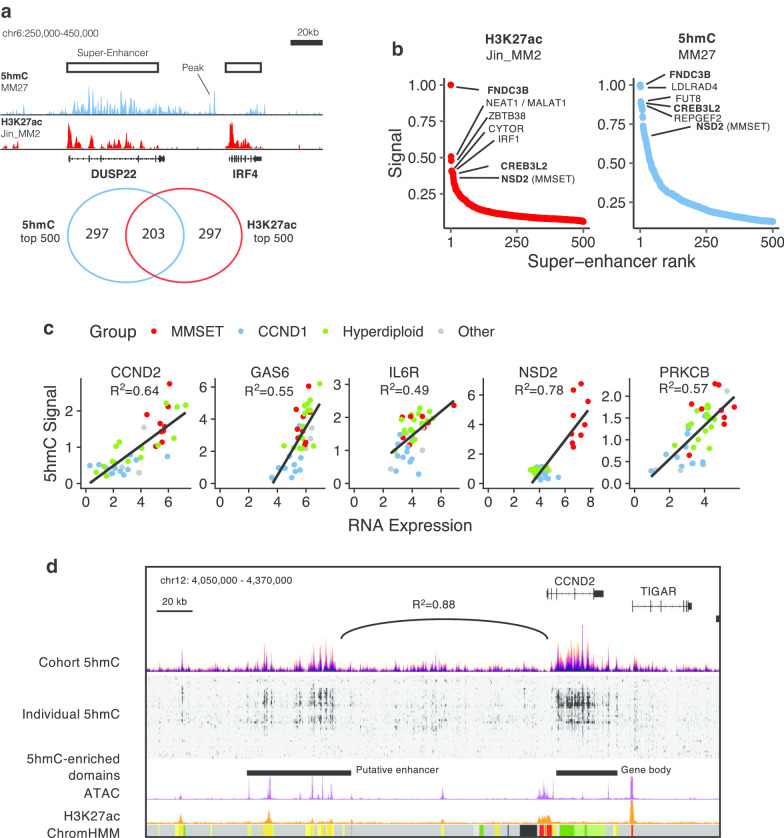
5hmC peaks are organized in large domains and correlate with RNA expression in MM. **a** Example of two 5hmC-enriched domains at the locus *DUSP22-IRF4* and Venn diagram showing the overlap between the top 500 5hmC-enriched domains (patient MM27) and H3K27ac super-enhancers (patient Jin_MM2). **b** ROSE plot (rank of super-enhancers) of the top 500 H3K27ac super-enhancers (patient Jin_MM2) and 5hmC-enriched domains (patient MM27). **c** 5hmC signal at 5hmC-enriched domains correlates with RNA expression of their neighbor gene. **d** 5hmC (this study), ATAC [33], H3K27ac [33] signals and ChromHMM states at the *CCND2* genomic locus. The 5hmC signal correlation between the *CCND2* gene and its putative enhancer is indicated

Similar to H3K27ac super-enhancers [37], we found that 5hmC is also organized in large peak clusters that we called 5hmC-enriched domains (Fig. 2a, b; Additional file 2: Figure S4B, Additional file 1: Table S2).

When we ranked 5hmC-enriched domains of a t(4;14) patient from IFM/DFCI and H3K27ac super-enhancers of a t(4;14) patient [33], we found an important overlap between genomic domains (Fig. 2a). In particular, *FNDC3B*, *CREB3L2* and *NSD2* are among the strongest common active genomic domains (Fig. 2b). To go further into the characterization of 5hmC landscape in NDMM, we applied the same procedure to each of the NDMM samples and we kept all the domains (*n* = 1816) overlapping in at least two samples. Interestingly, comparing with the literature, 41% of hypermethylated CpGs located in enhancer regions of B cells [26] were included within the 1816 5hmC domains, compared with only 11% (± 0.5%) when we chose random CpGs (Additional file 2: Figure S4C). This suggests that 5hmC persists at hypermethylated loci of MM.

Similar to H3K27ac super-enhancers, 5hmC levels in enriched domains may correlate with a proximal expressed gene (*R*^2^_max_ = 0.78, *n* = 1816, Additional file 1: Table S2). Briefly, 5hmc-enriched domains were associated with the most strongly correlated gene within its topologically associated domain (TAD) when we used TAD data from the GM12878 cell line (see Additional file 3: Methods). Remarkably, the strongest correlation between 5hmC and RNA expression is found at *NSD2* (*R*^2^ = 0.78), and highly expressed genes such as *CCND2* (*R*^2^ = 0.64), *GAS6* (*R*^2^ = 0.55), IL6R (*R*^2^ = 0.49) and *PRKCB* (*R*^2^ = 0.57) display high correlation coefficients with their neighboring 5hmC domain (Fig. 2c and Additional file 1: Table S2).

Several genes were found to have more than one neighboring 5hmC-enriched domain. In this case, two domains or more are found to correlate with the same proximal expressed gene (*i.e.,* genes *SECTM1* and *IQSEC1* have four 5hmC-enriched domains each; *ALOX5*, *GRIK4* have 3 neighboring domains; *CCND2*, *FRZB*, 2 domains; see Additional file 1: Table S2 for complete records). Finally, 1468 5hmC-enriched domains are specific to their putative gene.

Regardless of proximity to an expressed gene, we found the strongest correlation between two 5hmC-enriched domains at the CCND2 locus (*R*^2^ = 0.88) with 5hmC signal in *CCND2* gene body (hg38, chr12:4,278,700–4,312,900) and 5hmC signal at an extragenic domain located 120 kb upstream (hg38, chr12:4,106,500–4,164,700; Fig. 2d and Additional file 2: Figure S5A). Both 5hmC signals correlate strongly with *CCND2* RNA expression and both domains are located in the same topological domain according to HiC data [38] (hg38, chr12:3,850,000–4,800,000; Additional file 2: Figure S5B). The extragenic domain is also marked by ATAC and H3K27ac signals [33] in independent MM patients suggesting that this genomic domain is functionally active. This strongly suggests that this upstream domain is the enhancer of *CCND2* gene in the MM context.

We took advantage of the 5hmC-enriched domains and our RNA-seq data to search for core regulatory circuits as Saint-André and colleagues [39]. The concept of core regulatory circuits is based on the fact that only a small subset of interconnected TFs is responsible for the control of the whole transcriptome. We identified motifs associated with 39 TFs that bind 5hmC-enriched domains and regulate transcription of genes in the vicinity (Additional file 2: Figure S5C). Top expressed TFs binding 5hmC-enriched domains include *XBP1, ATF4, KLF6, USF2, IRF4, PRDM1, IRF1, KLF13, USF1* and *TCF3* (Additional file 1: Table S3).

### MM subgroups display specific 5hmC-enriched domains

**Fig. 3:**
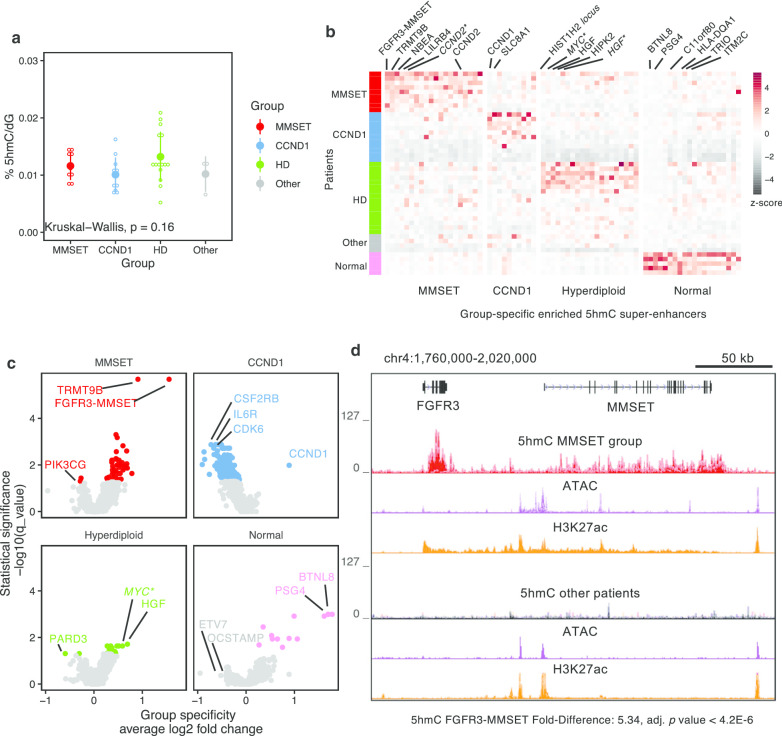
5hmC persists at specific oncogenic regions of myeloma subgroups. **a** MS quantification of 5hmC in myeloma cells of patients belonging to the MMSET (red), CCND1 (blue), hyperdiploid (HD, green) groups and the patients not related to any of the 3 groups (other, gray). **b** Heatmap of 5hmC-enriched domains specificity among the 40 MM patients and the 5 healthy donors (normal, pink). Color code for patients (in rows) corresponds to that of panel **a**. Asterisks stand for proximal non-genic loci. **c** Plot showing the 5hmC-enriched domains that are specifically enriched in patient groups MMSET, CCND1, hyperdiploid and normal plasma cells. Significant domains are colored. **d** 5hmC, ATAC and H3K27ac signals at the *FGFR3-MMSET* locus in the MMSET patient group and the non-MMSET patients

Patients’ samples were classified into 4 groups: MMSET [translocation t(4;14); 9 patients], CCND1 (RNA expression over 800 Transcripts per Million; 11 patients), hyperdiploid (16 patients; at least 2 odd chromosomal gains) and others (MM patients in none of the aforementioned groups; 4 patients). Global level of 5hmC measured by MS shows no significant difference between MM groups (Fig. 3a). However, locally, some 5hmC-enriched domains are found to be group specific (Fig. 3b, c, Additional file 2: Figure S6A and Additional file 1: Table S4). Remarkably, the strongest specific 5hmC-enriched domain for the group MMSET is overlapping the *FGFR3*-*MMSET* locus (*P* = 1.6E−6) followed by *CCND2*, *LILRB4*, *NBEA* and *TRMT9B* (Fig. 3d and Additional file 2: Figure S6B). This strong 5hmC enrichment in MMSET patients is also associated with strong H3K27ac and ATAC-seq signals in independent MM patients [33]. Again, in patients from the CCND1 group, the strongest and most specific 5hmC signal was located in the *CCND1* gene itself (Fig. 3c and Additional file 2: Figure S6C, *P* < 0.05). CCND1 group, which is determined by expression of CCND1 in absence of t(11;14) FISH, is marked by down-hydroxymethylation at *CSF2RB*, *IL6R* and *CDK6* loci (*P* < 0.05). The three genes are also downregulated in RNA-seq from the same patients’ samples (*P* < 0.01, data not shown).

The hyperdiploid group shows strongly specific 5hmC signal at *HGF* (hepatocyte growth factor, *P* = 0.02) and at the locus of *MYC* oncogene (Fig. 3b, c, Additional file 2: Figure S6D, *P* = 0.02). Normal plasma cells are enriched in 5hmC at *BTNL8*, *C11orf80*, *ITM2C*, *PSG4,* and *TRIO* genes (Fig. 3b, c). Of note, hematopoietic stem cell genes were not found over-represented when we performed a Gene Ontology term enrichment analysis. Main subgroups of NDMM thus show specific 5hmC at translocation breakpoints loci and at major oncogenes.

### 5hmC-enriched domains are dynamic between diagnosis and relapse in MM

**Fig. 4:**
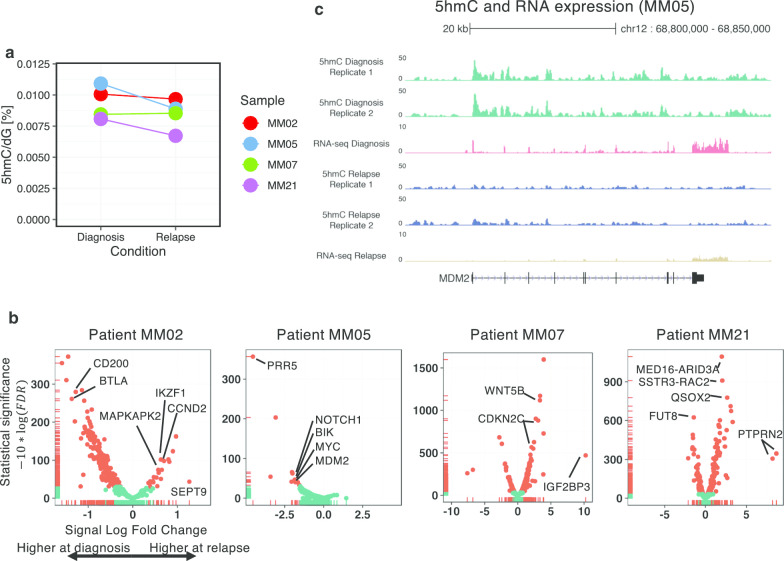
5hmC is dynamic and heterogeneous in MM between diagnosis and relapse. **a** MS quantification of 5hmC at diagnosis and relapse in patients MM02, MM05, MM07 and MM21 (average shown). **b** Plot showing the differential 5hmC-enriched domains between diagnosis and relapse in patients MM02, MM05, MM07 and MM21. **c** 5hmC signal and RNA expression levels of the *MDM2* gene at diagnosis and relapse in patient MM05. At relapse, myeloma cells harbor one copy of *TP53* locus (del17p FISH)

To identify the genomic domains associated with MM progression, we quantified and sequenced 5hmC genome-wide in four MM pairs (diagnosis and first relapse) and identified differential 5hmC-enriched domains using two replicates for each condition. CNV microarray data [40, 41] and RNA-seq were used at both time points to assess the progression of each patient. In three out of four paired samples, DNA hydroxymethylation slightly decreases at relapse (Fig. 4a). Although all patients’ samples display significant changes of 5hmC localization at relapse, there was no significant change in 5hmC that overlapped the four cases when we used DiffBind for differential enrichment analysis [42] (Additional file 1: Table S5). Since 5hmC landscape at relapse seems heterogeneous and patient-dependent, we focused our analysis on each of the four cases. Myeloma cells of patient MM02 showed a translocation of the *MMSET* locus [t(4;14)] and deletion of chromosomes 1p, 13 and 17p at diagnosis. This subject progressed in 18 months, with his myeloma cells displaying an additional third copy of the chromosome arm 1q. Out of 560 consensus 5hmC-enriched domains, 269 (48%) were significantly reduced at relapse compared to diagnosis (FDR < 0.05), while 20 (3.5%) increased at relapse. Interestingly, among the most significant gain of 5hmC-enriched domains, we found the *CCND2* and *IKZF1* gene bodies at relapse (respectively, 1, 6 and 1, fivefold; Fig. 4b) associated with a higher transcriptional activity (respectively, 2, 4 and 1, fivefold). It is interesting to note that the drug lenalidomide is effective by inducing IKZF1 proteasomal degradation via Cereblon [43, 44]. This suggests that the up-regulation of *IKZF1*, together with an increase in *CCND2* expression could favor disease progression. We also noticed a significant gain of 5hmC at *MAPKAPK2/MK2* at relapse. This gene is located on the chromosome arm 1 (gaining an extra-copy at relapse) and has been recently described as a poor prognosis factor in MM [45].

At diagnosis, patient MM05 displayed a classical hyperdiploid profile with amplification of chromosomes 3, 9, 11, 15, 19, in which we found 675 5hmC-enriched domains. At relapse 24 months later, myeloma cells displayed a 1-copy loss of the chromosome arm 17p and 22 5hmC-enriched domains (3.2%) significantly decreased. In particular, we found a lower expression and lower 5hmC signal at TP53 target gene *MDM2* (Fig. 4b, c).

Patient MM07 is another *MMSET* translocated patient [t(4;14)] with amplification of the chromosome arm 1q and deletions of chromosomes 13 and 17p (Fig. 4b) at diagnosis. At relapse, there are additional deletions of chromosomes arms 3p, 6q and 8q, and 5hmC-enriched domains increase at several genes including *CDKN2C*, *IGF2BP3* and *WNT5B* as well as their RNA expression (logFC > 3, Additional file 2: Figure S7).

Patient MM21 plasma cells were found to have a narrow *TET2* deletion at diagnosis, but surprisingly 2 heterozygous copies of *TET2* at relapse. TET2 protein, which oxidizes 5mC in 5hmC, is frequently loss-of-function-mutated in cancer, especially in myeloid malignancies [46], but not in MM [32]. A total of 385 domains were found, out of which 86 (22%) decreased and 117 (30%) increased at relapse and global level of 5hmC decreased despite the reappearance of the missing *TET2* copy.

These data indicate that 5hmC signal varies between diagnosis and relapse in a patient-specific manner. However, several genes and enhancers, whose activities changed at relapse, could be identified as putative drivers of disease progression.

## Discussion

We have shown that the epigenetic mark 5hmC is lower in MM than in normal plasma cells, and that it gradually decreases with the disease severity of patients while being independent of patient’s age. Our observation corroborates the theory of DNA hydroxymethylation being linked to mitotic index [13, 14] as 5hmC decreases with the tumor severity. In that sense, global levels of hydroxymethylation are consistent with the already-described global hypomethylation of MM. With a limited number of samples (*N* = 39) and events (*N* = 16), 5hmC level tends to correlate with longer survival (*P* < 0.1). This suggests that the 5hmC level is a prognosis biomarker for newly diagnosed MM. Independent cohort followed on a longer time will be needed to confirm the good-prognosis association that we observe. In any case, our study supports the potential use of 5hmC and 5mC quantification as a clinical biomarker in MM.

Our analysis shows that 5hmC localizes predominantly at transcriptionally active regions. Surprisingly, this is true despite a global and important loss of 5hmC. The mark seems to be maintained by TET proteins at highly active chromatin. This stays consistent with the fact that the DNA methylation in MM is reduced globally but maintained at intronic enhancers regions [26]. In a sense, there is an association between the remaining localization of 5mC and 5hmC in MM, although the marks are thought to flag different regions: inactive versus active chromatin.

Given that 5hmC efficiently marks active chromatin, we applied the H3K27ac super-enhancer discovery algorithms to the 5hmC signal and found similar regions. This analysis highlighted key components of the plasma cell biology (*CREB3L2* for example), and more importantly, of myeloma subgroups such as *MMSET* locus in t(4;14) patients. We show that defining 5hmC-enriched domains similar to H3K27ac super-enhancers, rather than studying single 5hmCpGs, is relevant and powerful to decipher major disease drivers on patient’s genomic DNA.

We also show that major well-known translocation events produce massive oxidation at the recombinant loci, together with previously shown chromatin opening and high transcription level. In addition to translocation and myeloma subgroups, we found novel active domains, for instance, proximal to *CCND2* and *TRMT9B* in MMSET patients. De novo combined analysis of 5hmC and RNA expression revealed key transcription factors involved in the disease such as IRF4, PRDM1 and TCF3. These TFs likely drive the malignant transcriptome as they are essential in most of the MM cell lines in CRISPR KO screening from DEPMAP (https://depmap.org/portal/). Despite being difficult to drug, we believe that these TFs are therapeutic targets of interest.

Between diagnosis and relapse, we found a highly dynamic and patient-specific distribution of the 5hmC signal. This reflects that MM progression is highly heterogeneous, although we could find some consistency between 5hmC changes, RNA expression and copy number variations. In the future, it would probably be more meaningful to describe the dynamics of 5hmC on more samples and at several time-points of the disease progression.

Profiling chromatin with histone marks (*e.g.,* H3K27ac ChIP-seq) or chromatin accessibility (ATAC-seq) requires nuclei and thus limits the study of epigenetics on clinical cohorts with only genomic DNA stored for historical and practical reasons. The technical limitation of preserving frozen nuclei is overcome by mapping active domains of chromatin directly on genomic DNA through the 5hmC mark produced by the TET proteins. Our study shows indeed that the epigenetic mark 5hmC is valuable to discover active regulatory domains in genomic DNA from a cohort of patients without the need of chromatin extraction. Furthermore, it has been recently shown that it is also possible to map 5hmC on circulating DNA [18, 19]. This makes 5hmC not only biologically valuable, but it is also technically easier to map genome-wide or at key oncogenic loci. Taken together, these results show the value of epigenomics in retrospective studies and bring to light potential drug targets that drive the malignant transcriptome.

## Conclusions

We show that DNA hydroxymethylation is an active chromatin mark that is globally depleted in malign plasma cells but remains at MM essential genes that drive the malignant transcriptional network. Furthermore, the global DNA hydroxymethylation level decreases with MM severity and tends to be associated with outcome. This observation needs to be further assessed. Remaining hydroxymethylation localizes at major translocation breakpoints and active chromatin. It is thus a potent mark to discover oncogenic drivers of the disease. We expect DNA hydroxymethylation, and DNA modifications in general, to become major clinical biomarkers in the future, especially when direct-DNA-sequencing will be efficient and cost-effective, allowing the genome-wide mapping of DNA modifications in a single run.

## Methods

### Genomic DNA extraction of normal plasma cells and multiple myeloma cells

Normal plasma cell and multiple myeloma cells collection and purification from human bone marrow are described in Additional file 3: Methods. Genomic DNA was extracted with Qiagen Allprep DNA/RNA Mini Kit (ref. 80204). DNA samples were dosed by DNA HS QuBit and the absence of contaminant RNA checked by RNA HS Qubit.

### Patients selection for this study

No statistical method was used to predetermine sample size. In this study, we have selected 40 patient samples from the IFM/DFCI cohort (NCT01191060) further detailed in Additional file 3: Methods. Samples were selected with: low level or absence of RNA in DNA samples, low level of rRNA in RNA-seq data, enough DNA material available and high percentage of CD138+ cells (98% in average in this study). Healthy individuals and myeloma patients are of comparable age (resp. 62 and 58 in median). Men were overrepresented in the healthy group (*N* = 4/5). Neither control individuals nor myeloma patients were given vitamin C supplementation; however, nutritional aspects have not been checked before patient selection [47]

### Digestion of genomic DNA and subsequent LC–MS analysis

The genomic levels of 5mC and 5hmC were quantified using a mass spectrometry-based stable isotope-dilution method [48]. For each LC–MS measurement, 70 ng of genomic DNA was digested to the nucleoside level. As heavy-atom-labeled internal standards, fixed quantities of D_3_-5mC and D_2_^15^N_2_-5hmC were added to the mixture. For each biological sample, two independent measurements (technical replicates) were taken. Quantitative LC–ESI–MS/MS analysis was performed using an Agilent 1290 UHPLC system coupled to an Agilent 6490 triple quadrupole mass spectrometer in conditions similar to Traube et al. [48]. Further details are described in Additional file 3: Methods.

### Selective chemical labeling of 5hmC coupled with sequencing (5hmC-seq)

For each sample, 550 ng of genomic DNA was sonicated with a Bioruptor Pico in Tris 10 mM pH 8 to obtain DNA fragments of 300 bp in average. 25 pg of 5hmC control spike-in was added to the sonicated DNA (control provided by the kit HydroxyMethyl Collector, Active Motif, ref. 55013). 50 ng of DNA was conserved at this stage to make the input library later. The remaining DNA was processed using the HydroxyMethyl Collector kit (method from Song et al. [35]) to glycosylate and biotinylate specifically the genomic 5hmC. After glycosylation and biotinylation, the DNA was purified with Ampure beads (Beckman Coulter, ref. A63881). The DNA fragments containing the biot-glu-5hmC were purified with Streptavidin beads (Active Motif, ref. 55013), eluted, purified with Ampure beads and finally eluted in 50 uL Tris pH 8. The 5hmC-seq libraries were prepared with the kit NEBNext Ultra II DNA library prep kit for Illumina (ref. E7645S) and indexed with NEBNext dual indexed primers (E7600S). The libraries were quality-checked by HS DNA Agilent BioAnalyzer (Additional file 2: Figure S1), dosed by DNA HS Qubit, pooled and submitted to the genome sequencing platform for Single-Read 50 bp Illumina HiSeq-2500 Rapid Run sequencing.

### RNA-seq libraries

As the IFM-DFCI did not include RNA-seq data from patients at relapse, we produced additional RNA-seq data at diagnosis and at relapse for 4 MM patients (patient number MM02, MM05, MM07 and MM21). The RNA-seq libraries were prepared using the NEBNext Poly(A) mRNA Magnetic Isolation Module (NEB, ref. E74905) and the NEBNext Ultra II Directional RNA Library Prep Kit for Illumina (NEB, ref. E7760S) and sequenced by an Illumina Rapid Run HiSeq 2500 Single-Read 50 bp.

*Bioinformatics* (cf. Additional file 3: Methods).

## Supplementary information

**Additional file 1**. **Figure S1**: Agilent BioAnalyzer profile of a 5hmC-seq Illumina library. **Figure S2**: MS quantification of 5mC in genomic plasma cell DNA is independent of age and sex. **(A)** Dot plot of 5mC global quantification by MS in normal plasma cells from healthy donors (N=5), and of myeloma cells of patients at diagnosis (N=40). **(B)** Dot plot of 5mC global quantification by MS by disease stage (ISS I N=9; ISS II N=17; ISS III N=13; NA=1). 5mC **(C)** and 5hmC **(D)** dot plot of MS quantification depending on the sex of the patients. 5mC **(E)** and 5hmC **(F)** dot plot of MS quantification depending on the age of the patients. **Figure S3**: Survival course depending on DNA methylation (5mC) level-based separation of two risk groups of NDMM (n=20 and 20). **Figure S4**: 5hmC association with expression and criteria of 5hmC peaks to merge in 5hmC peak clusters. **(A)** Average level of 5hmC in all genes normalized to the same body length. Red line stands for average 5hmC in genes with high expression level (greater than 100 Reads per Kilobase Million (RPKM)). Orange line stands for medium expression level (between 10 and 100 RPKM). Green line represents lowly expressed genes (between 1 and 10 RPKM), and blue line stands for very lowly expressed genes (below 1 RPKM). **(B)** Stitching of 5hmC into 5hmC-enriched domains. The y-axis represents the number of peaks left after merging. The x-axis represents the distance between peaks to merge. Each 5hmC sample was analyzed (one color per patient). The distance 12.5 kb was chosen to stitch 5hmC peaks into the 5hmC-enriched domains that we describe in this study. **(C)** Fraction of overlap between 5hmC-enriched domains of this study and CpG from the Illumina 450K chip. The red bar represents overlap with hypermethylated CpGs in B cell-specific enhancers that were described by Agirre and colleagues (see Additional file 3: Methods). Blue bars represent random CpGs from the same chip. **Figure S5**: 5hmC allows the identification of a putative CCND2 enhancer. **(A)** Correlation between CCND2 expression, 5hmC at CCND2 gene body and 5hmC at the putative 5hmC enhancer across the 40 MM patients. **(B)** Hi-C contact map in lymphoblastoid cells (GM12878 cell line) at the CCDN2 locus showing the spatial interaction between CCND2 gene and its putative enhancer. **(C)** Expression of core transcription factors predicted to orchestrate core regulatory circuitries with 5hmC and RNA expression genomic data. **Figure S6**: MM 5hmC-enriched domains associate with H3K27ac super-enhancers. Rank ordering of the 100 strongest 5hmC-enriched domains on average in the cohort **(A)**, in the MMSET group **(B)**, in the CCND1 group **(C)** and in the hyperdiploid group **(D)**. Color highlights domains present in only one of the ROSE plots by group. **Figure S7**: 5hmC signal levels at WNT5B-associated domain are increased at relapse in MM07. **(A)** Normalized 5hmC enrichment at WNT5B-associated domain. Point shapes match replicates. Fold change=1.3, p=0.003, FDR>0.1. **(B)** Gene expression levels in RPKM measured by RNA-seq at diagnosis and relapse for three genes surrounding the WNT5B-associated domain. **(C)** 5hmC genomic signal around WNT5B-associated domain. Colors match those of (A) and (B). 5hmC domain is depicted under signal tracks (hg38: chr12:1,517,750-1,621,200).

**Additional file 2**. **Table S1**: Patients characteristics, survival and mass spectrometry quantification of 5hmC and 5mC. **Table S2**: Scoring of 1816 5hmC-enriched domains across NDMM samples. **Table S3**: Motif analysis of core regulatory circuitries. **Table S4**: Scoring of groups-specific 5hmC-enriched domains. **Table S5**: Differential 5hmC-enriched domains between at diagnosis and relapse (DiffBind analysis).

**Additional file 3.** **Methods**: This file describes normal plasma cells purification, myeloma cells purification, 5mC and 5hmC dosage by mass spectrometry, bioinformatics methods and statistical analysis.

## Data Availability

Sequencing data are accessible at European Nucleotide Archive under accession number PRJEB32800. Mass spectrometry data are available in additional file table. Bioinformatics code is available on request.

## Abbreviations

5caC: 5-CarboxylCytosine
5fC: 5-FormylCytosine
5hmC: 5-HydroxymethylCytosine
5hmCpG: 5′-HydroxymethylCytosine-phosphate-Guanine-3′
5mC: 5-MethylCytosine
ATAC: Assay for transposase-accessible chromatin
ChIP: Chromatin immunoprecipitation
CI: Confidence interval
CNV: Copy number variation
CpG: 5′-Cytosine-phosphate-Guanine-3′
DNMT1 (3A/3B): DNA (cytosine-5)-methyltransferase 1 (3A/3B)
FISH: Fluorescence in situ hybridization
HDAC: Histone deacetylase
HR: Hazard ratio
ISS: International Staging System
LC–MS: Liquid chromatography–mass spectrometry
MM: Multiple myeloma
MAPK: Mitogen-activated protein kinases
MS: Mass spectrometry
NDMM: Newly diagnosed multiple myeloma
NF-κB: Nuclear factor-kappa B
NPC: Normal plasma cell
PC: Plasma cell
ROSE: Rank of super-enhancers
RPKM: Reads per kilobase of transcript million mapped reads
rRNA: Ribosomal RNA
TAD: Topologically associated domain
TET1 (2/3): Ten-eleven translocation methylcytosine dioxygenase 1 (2/3)
TFs: Transcription factors
TSS: Transcription start site
