# Supplementary material for: DNA hydroxymethylation is associated with disease severity and persists at enhancers of oncogenic regions in multiple myeloma
Source: Clin Epigenetics. 2020 Nov 2;12:163. doi: 10.1186/s13148-020-00953-y (PMC7607866; doi:10.1186/s13148-020-00953-y)

## Supplementary Figure 1

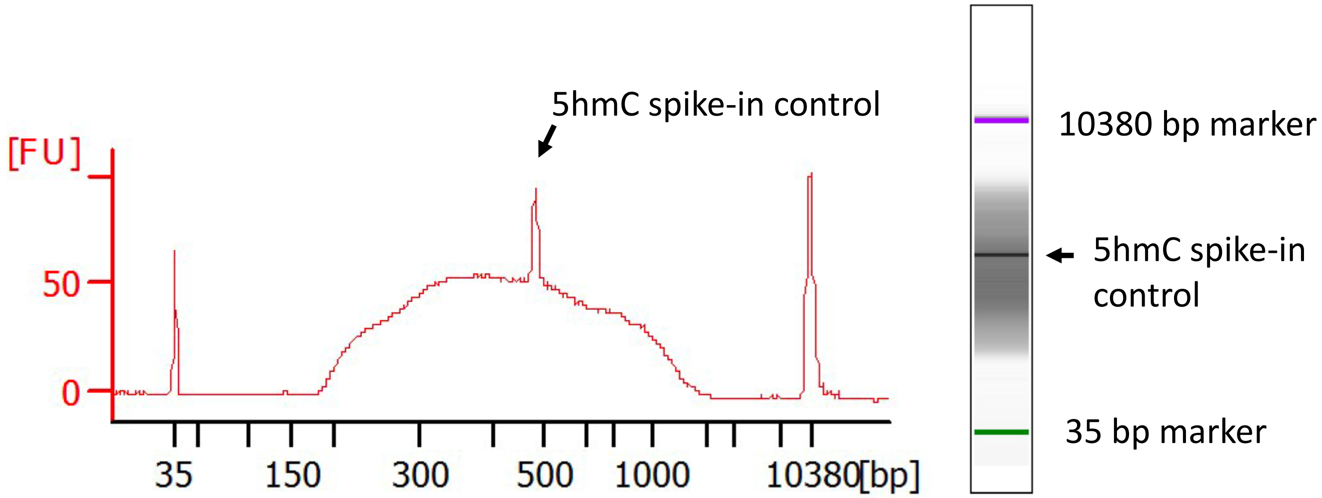

# Supplementary Figure 2

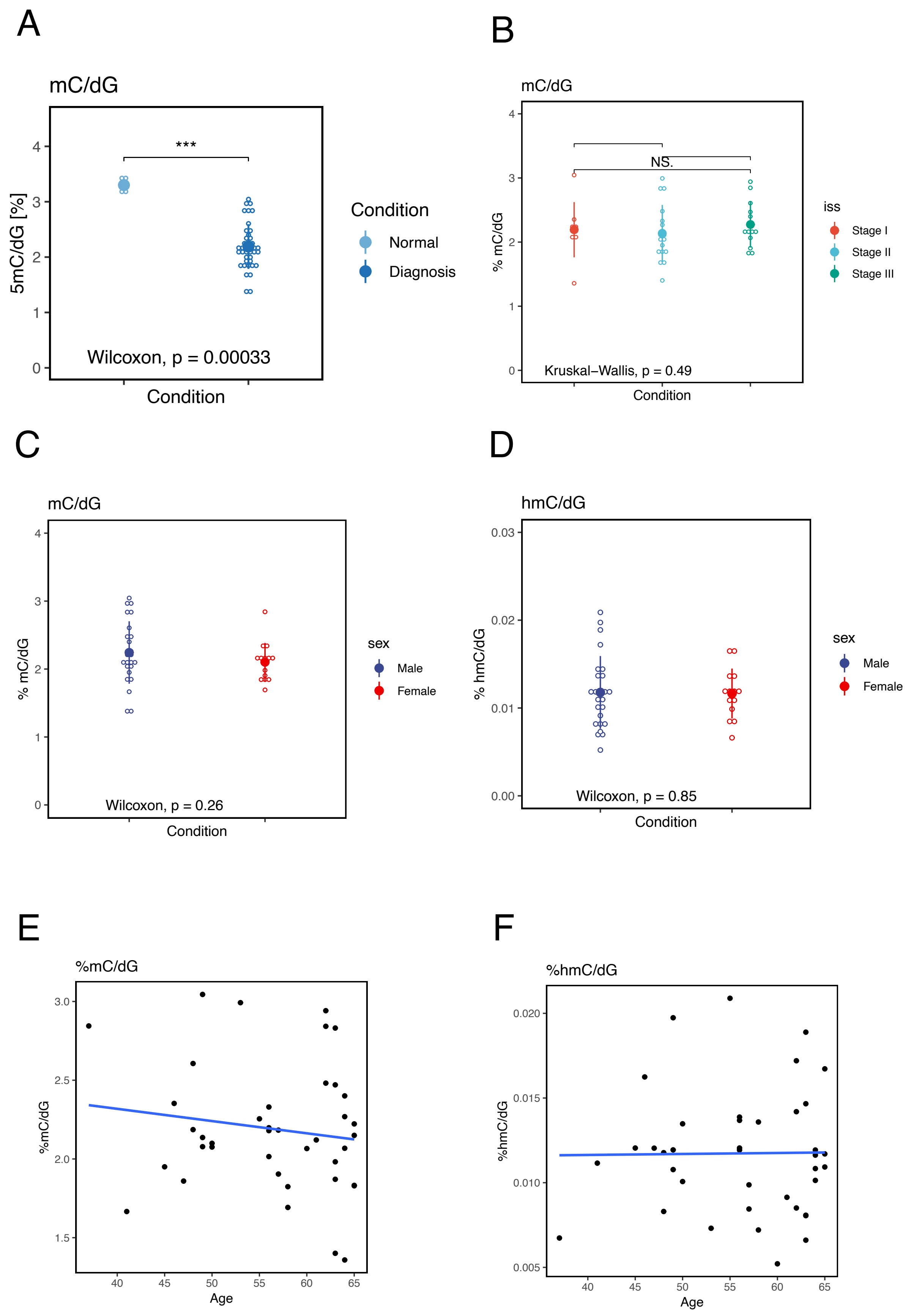

# Supplementary Figure 3

A

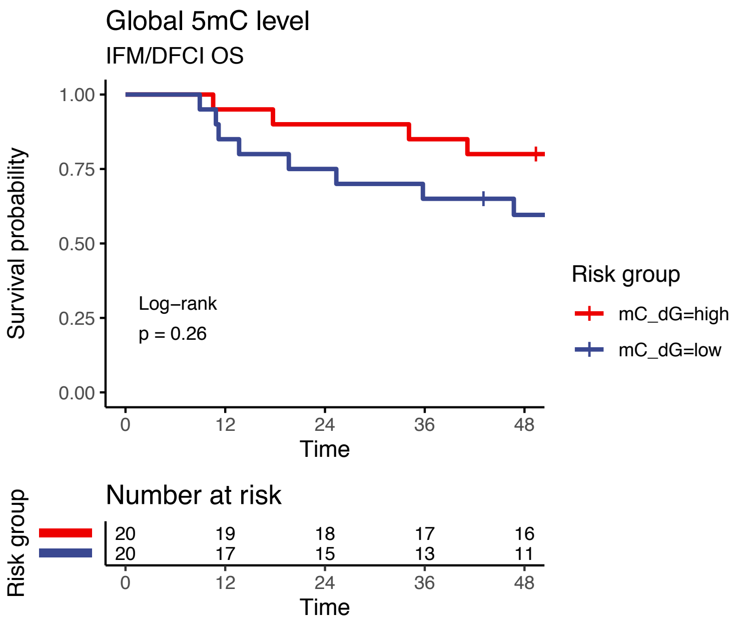

# Supplementary Figure 4:

A

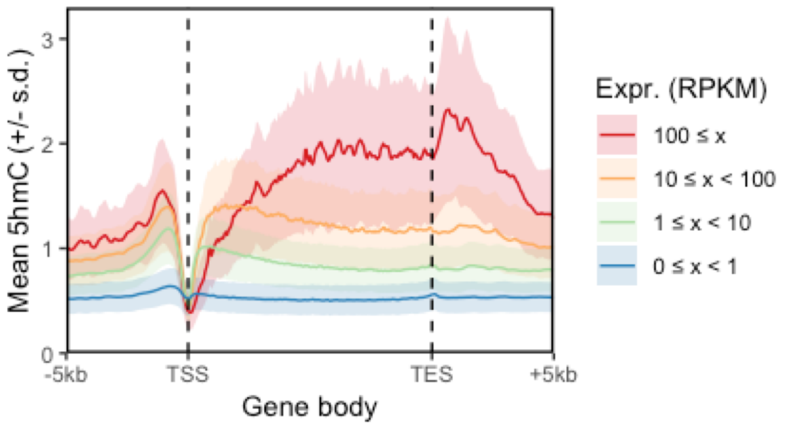


B

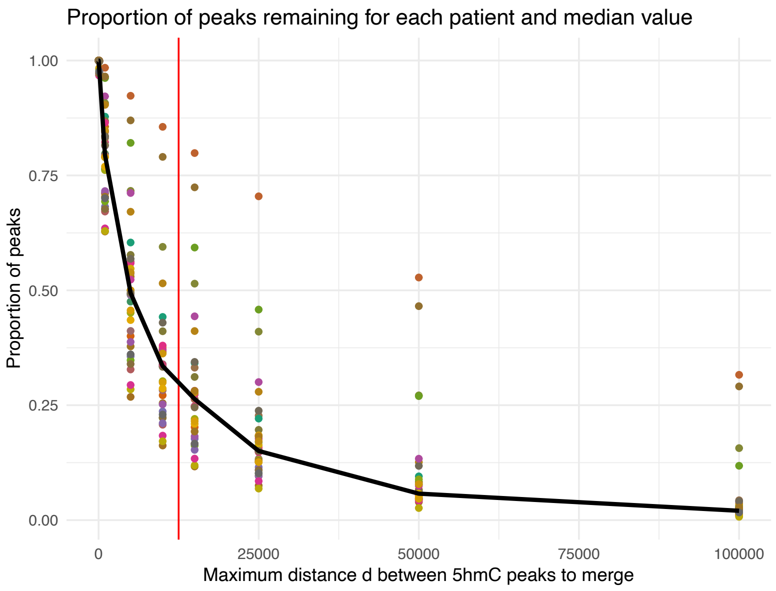


C

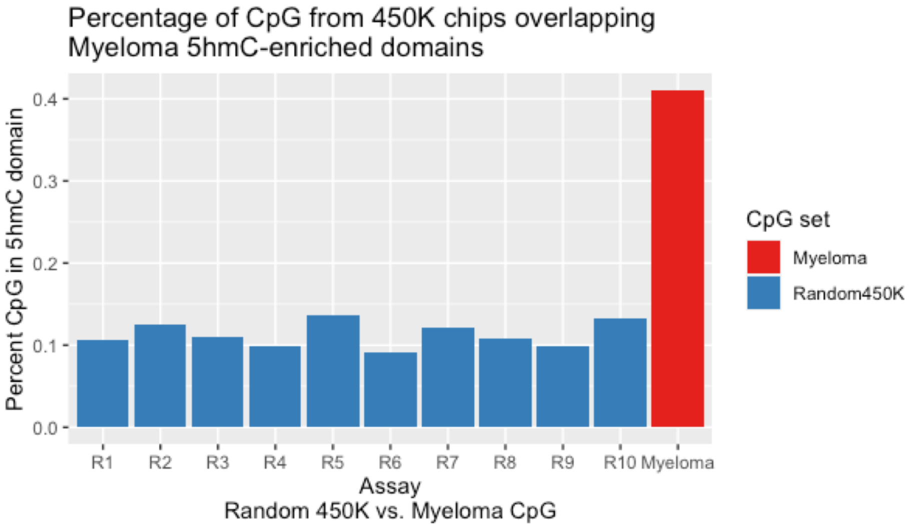

# Supplementary Figure 5

A

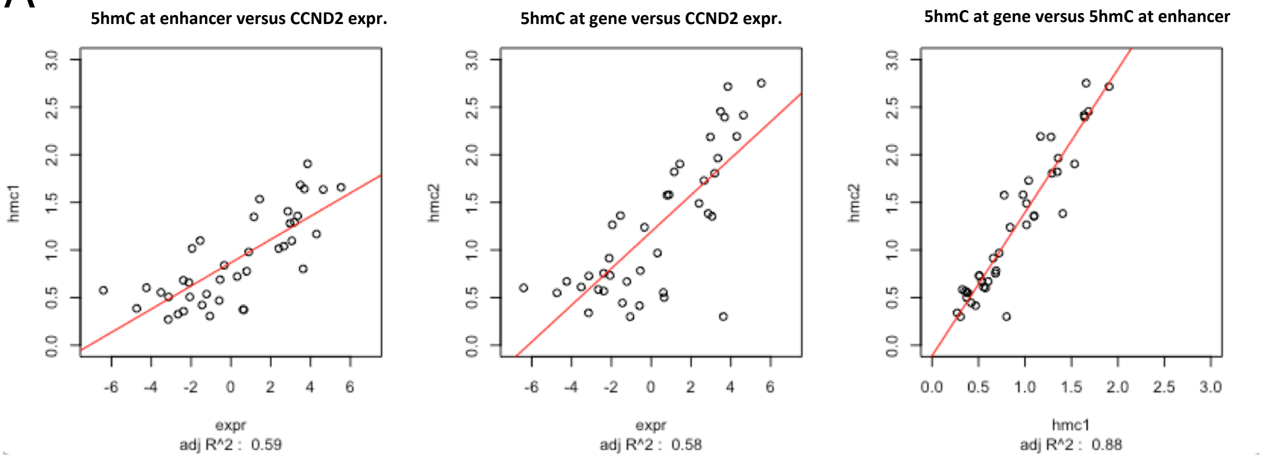


B

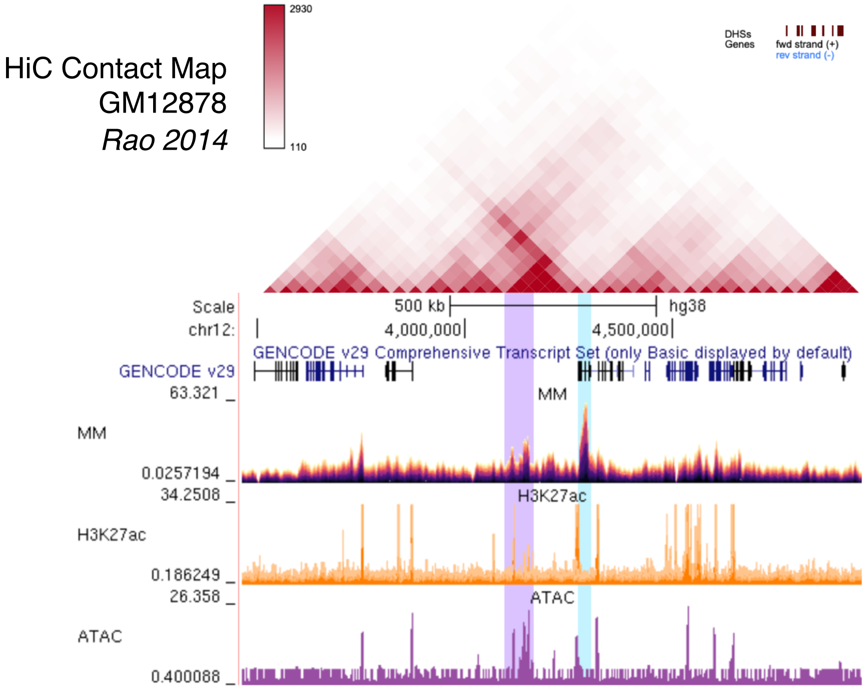


C

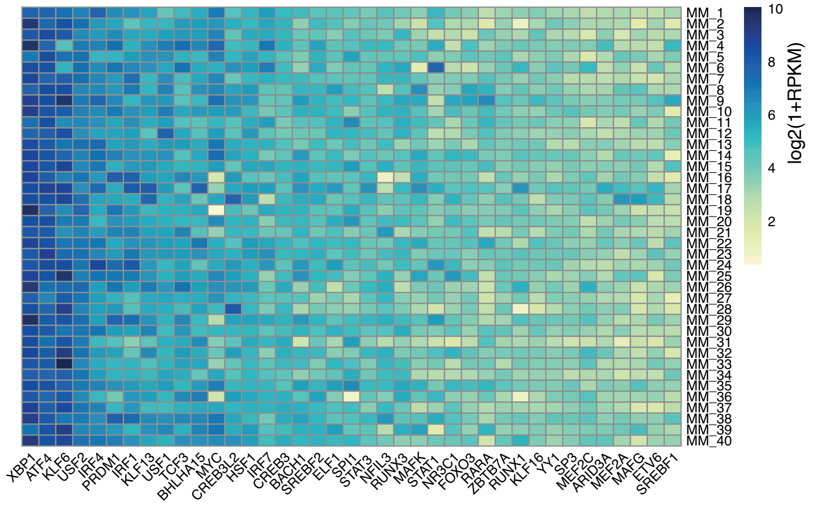

# Supplementary Figure 6

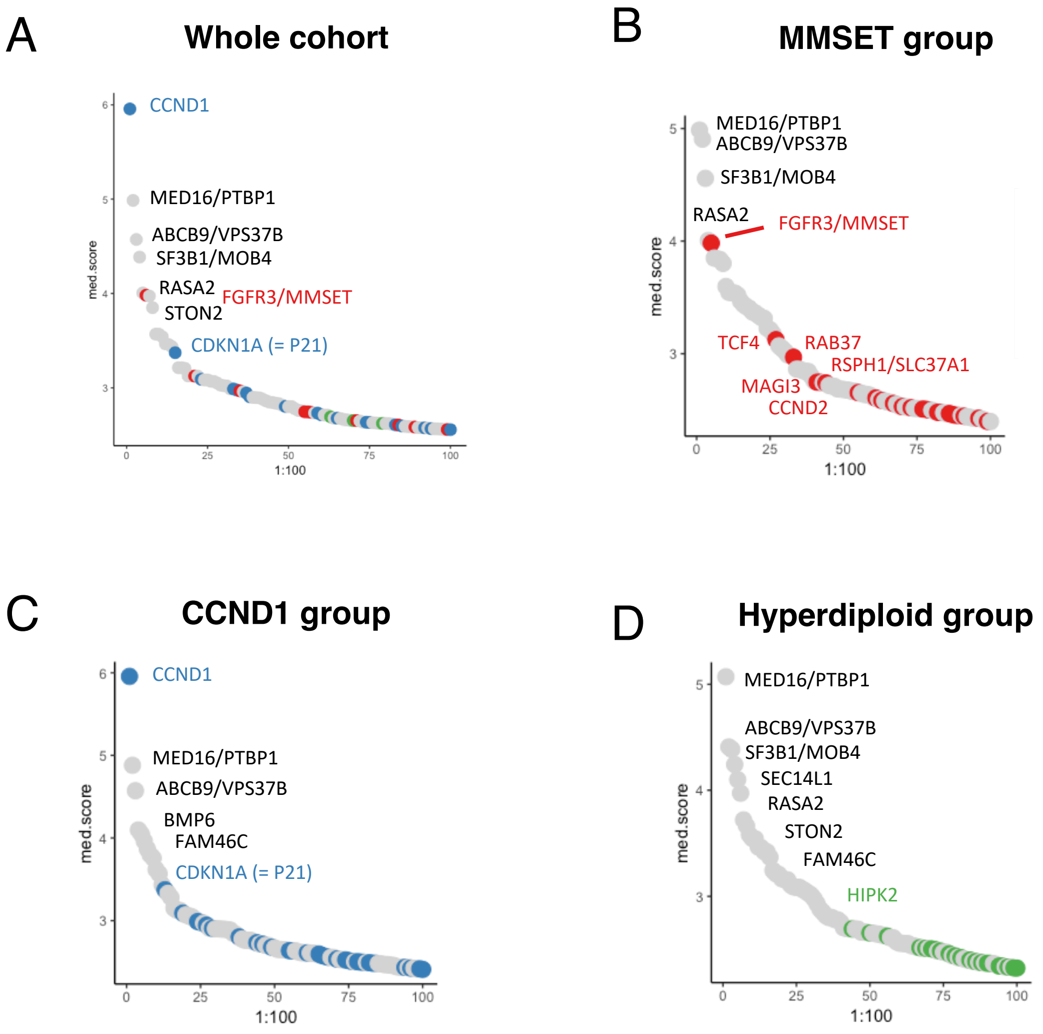

# Supplementary Figure 7

A

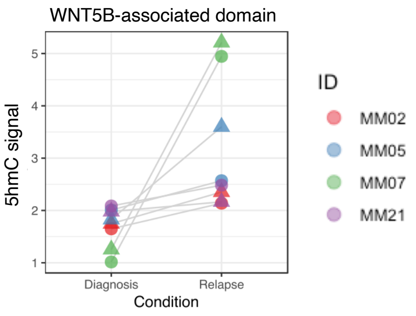


B

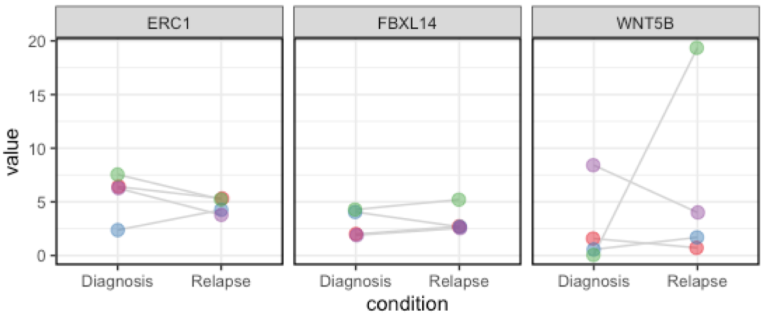


C

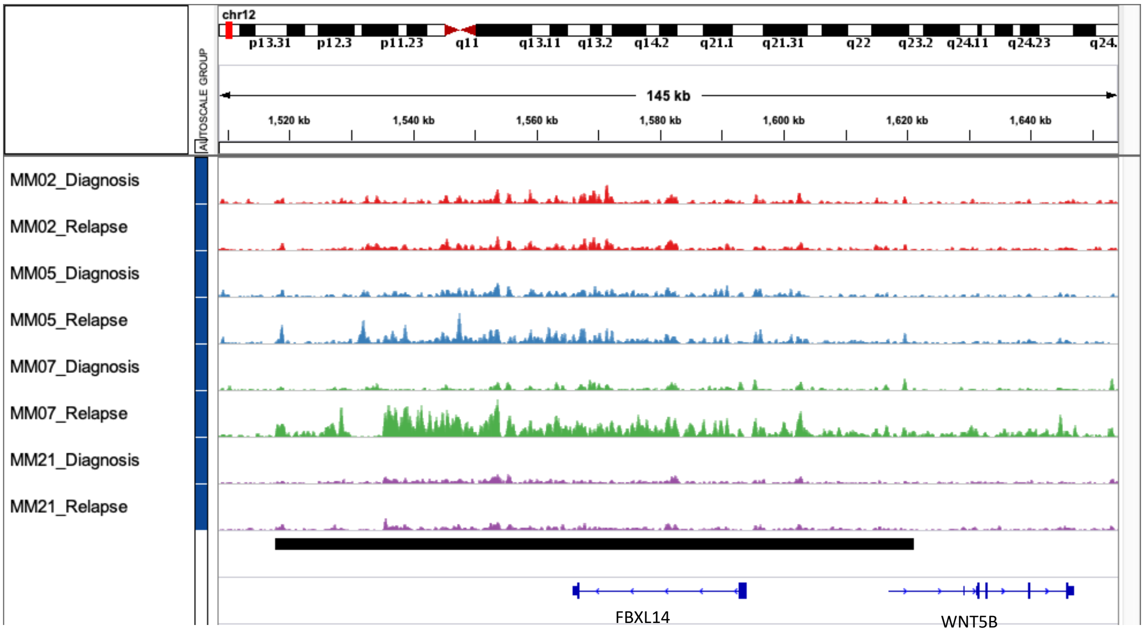

Supplement: Supplementary file 2 — Additional file 2. Table S1: Patients characteristics, survival and mass spectrometry quantification of 5hmC and 5mC. Table S2: Scoring of 1816 5hmC-enriched domains across NDMM samples. Table S3: Motif analysis of core regulatory circuitries. Table S4: Scoring of groups-specific 5hmC-enriched domains. Table S5: Differential 5hmC-enriched domains between at diagnosis and relapse (DiffBind analysis). [file 13148_2020_953_MOESM2_ESM.pdf]
