# Supplementary material for: DNA hydroxymethylation is associated with disease severity and persists at enhancers of oncogenic regions in multiple myeloma
Source: Clin Epigenetics. 2020 Nov 2;12:163. doi: 10.1186/s13148-020-00953-y (PMC7607866; doi:10.1186/s13148-020-00953-y)
Supplement: Supplementary file 3 — Additional file 3. Methods: This file describes normal plasma cells purification, myeloma cells purification, 5mC and 5hmC dosage by mass spectrometry, bioinformatics methods and statistical analysis. [file 13148_2020_953_MOESM3_ESM.docx]

# SUPPLEMENTS

# SUPPLEMENTARY METHODS

## NORMAL PLASMA CELLS PURIFICATION

After informed consent, the femoral canal of individuals with isolated hip osteoarthritis who were otherwise healthy was probed with a metal suction device following femoral neck removal. Bone marrow cells were suctioned into a tube that contained EDTA, placed on ice and immediately transported to our laboratory. BMMCs were purified by Ficoll. Normal plasma cells were FACS-sorted using a BD FACSAria III as CD38/CD138 positive and CD3/CD13/CD33 negative (antibodies from Becton Dickinson, ref. 345807, 555332, 555394, 555450 and 562935).

## MYELOMA CELLS PURIFICATION

Bone marrow biopsies were realized on MM newly diagnosed patients from cohort IFM-DFCI 2009 (Intergroupe Francophone du Myélome - Dana Farber Cancer Institute)^1,2^. All patients signed an informed consent form approved by the Toulouse Ethics Committee. All included patients were newly diagnosed with symptomatic MM based on International Myeloma Working Group 2003 Diagnostic Criteria^1^. All the samples were collected in France and processed at the University Hospital of Nantes. Bone Marrow Mononuclear Cells (BMMCs) were purified by Ficoll. Plasma cells were purified with anti-CD138 beads (Robosep platform, StemCell Technologies) and the CD138-positive percentage of cells was checked by immunofluorescence microscopy.

## 5MC AND 5HMC DOSAGE BY MASS SPECTROMETRY

The genomic levels of 5mC and 5hmC were quantified using a mass spectrometry-based stable isotope-dilution method^3^. For each LC-MS-measurement (technical replicate), 70 ng of genomic DNA (gDNA) were digested to the nucleoside level using the Nucleoside Digestion Mix (ref. M0649S) from New England BioLabs. To this reason, a solution of 70 ng gDNA in 38 $\mu$L of milliQ-water was prepared. As heavy-atom-labeled internal standards, 1.28 pmol D_3_-5mC and 0.193 pmol D_2_ ^15^N_2_-5hmC in 6 $\mu$L of milliQ-water were added to the solution, followed by 5 $\mu$L of the Nucleoside Digestion Mix Reaction Buffer (10x), and 1 $\mu$L of the Nucleoside Digestion Mix. After incubation for 90 min at 37C, the mixture was filtered using an AcroPrep Advance 96 filter plate 0.2 $\mu$m Supor from Pall Life Sciences and subsequently analysed by LC-MS. For each biological sample, two independent measurements (technical replicates) were performed. Quantitative LC-ESI-MS/MS analysis of the enzymatically digested DNA samples was performed using an Agilent 1290 UHPLC system coupled to an Agilent 6490 triple quadrupole mass spectrometer. The UHPLC-conditions used and the settings of the mass spectrometer were the same as previously published by Traube and colleagues^3^.

## AVAILABILITY OF DATA

Sequencing data are accessible at European Nucleotide Archive under accession number PRJEB32800. Mass spectrometry data are available in Supplemental Table. Bioinformatics code is available on request.

### BLUEPRINT DATA STATEMENT

This study used data generated by the BLUEPRINT Consortium. A full list of the investigators who contributed to the generation of the data is available from www.blueprint-epigenome.eu. Funding for the project was provided by the European Union’s Seventh Framework Program (FP7/2007-2013) under grant agreement no 282510 – BLUEPRINT.

### BIOINFORMATICS

### 5hmC-seq analysis

5hmC-seq reads were trimmed with Cutadapt v1.18^4^ and mapped to human reference genome GRCh38 using Bowtie2 v2.2.8^5^ with default parameters. Duplicates and reads of low mapping quality ($Q<30$) were discarded. 5hmC peak clusters were defined similar to Boeva and colleagues^6,7^ with a stitching distance of 12.5kb. Blacklisted regions consist of chromosomes X, Y, ENCODE reference blacklist and peaks called on input-seq libraries. Peak calling was done with HMCan^6^ with default parameters. 5hmC peaks were then merged using LILY^7^ at a distance of 12.5 kb to produce what we call 5hmC-enriched domains. The value of 12.5 kb is the same as that of Boeva and colleagues (Supplementary Figure 2) and global 5hmC signal was then re-normalized^7^. We defined a set of consensus peaks clusters by merging 5hmC-enriched domains and keeping arbitrary those occupied by at least two samples across the cohort. When specified, the process was applied to the cohort and normal plasma cells together. We excluded regions shorter than 12 kb. Average 5hmC signal depth over consensus peaks clusters was computed for each sample to obtain the 5hmC score matrix $M_{i,j}$ where $i$ is a sample and $j$ a consensus peak cluster.

Genes and 5hmC peaks clusters were associated to the Topologically Associated Domain (TAD) of the B-cell derived cell line GM12878 they are located in similar to Boeva and colleagues^7^.

Group specific 5hmC peaks clusters were identified by computing the average Log2 Fold-Change signal per region between groups and tested with a Wilcoxon rank-sum test adjusted with a Benjamini-Hochberg correction. For paired samples (two replicates of each diagnosis and relapse), we used DiffBind^8^ with default parameters to find differentially enriched 5hmC regions.

Potential core regulatory circuitries were found with CRCMapper^9^ using default parameters.

Overlap between 5hmC-enriched domains and previously published hypermethylated CpGs in MM^10^ was computed with R and compared to overlap with random CpGs obtained from the Illumina Methyl450K table in UCSC (<https://genome.ucsc.edu/cgi-bin/hgTables>).

Gene Ontology enrichment analysis was performed with GSEA^11^ and the 100 first gene sets with FDR<0.05 were inspected. The following Gene Ontology IDs were kept for hematopoietic stem cell gene enrichment analysis: GO: 0060218, GO:0061484, GO:0035701, and GO:0071425.

### Chromatin states annotation with ChromHMM

Functional annotation was done with ChromHMM^12^ based on modified histone ChIP-seq data from the cell lines MM.1S, NCI-H929 and GM12878. Individual track of NCI-H929 was kept for statistical analysis. We downloaded the ChIP-seq data from the ENCODE portal^13,14^ (<https://www.encodeproject.org/>). The 105 libraries identifiers used can be communicated upon request.

### RNA-seq analysis

IFM-DFCI RNA-seq data were obtained from Cleynen and colleagues^15^ or generated by us. RNA-seq libraries were trimmed using Cutadapt v1.13^4^ with parameter $-m 20$ and aligned to hg38/GRCh38 using STAR v2.5.3a^16^ with default parameters. Genes were quantified using featureCounts^17^ from the R package Rsubread and the Gencode v28 genome annotation.

### ChIP-seq and ATAC-seq analysis

ChIP-seq and ATAC-seq libraries were downloaded from the European Nucleotide Archive (project number PRJEB25605^18^) or from Blueprint Epigenome project and were analyzed with the pyflow-ChIPseq^19^ and pyflow-ATACseq pipelines respectively with default parameters. Chromatin-states genome annotation was realized using ChromHMM^12^ on ChIP-seq data from NCI-H929 cell line.

### Statistical analysis

Quantitative bio-clinical variables were described with median and interquartile range (IQR) or mean and standard deviation. Significance of mean difference between groups was assessed with Kruskal-Wallis method for multiple group testing, and/or Wilcoxon test for 2 groups. Qualitative variables were described using frequency of their respective modalities. Significance of heterogeneity of the distribution between groups was assessed with $\chi^{2}$ Pearson’s test (or Fisher’s exact test if appropriate). DESeq2 FDR testing was used for diagnosis-relapse expression and 5hmC testing. Signal correlation was assessed with a Spearman correlation. Otherwise, Pearson correlation was used. For survival analysis, time-to-event was calculated from the randomization to the event date, *i.e.* death for OS, or to the last follow-up date. For MS quantitative variables, cohorts were then split at the median value of high and low 5hmc (and 5mC). Hazard ratio between groups were calculated using Univariate Cox model. Survival curves were calculated using the Kaplan-Meier method and groups were compared using a Log-rank test. P values were corrected for multiple testing with the Benjamini-Hochberg method. Adjusted p values under 0.05 were considered significant. All calculations were done using R 3.5.0 software.

**REFERENCES OF SUPPLEMENTARY METHODS**

1. The International Myeloma Working Group*. Criteria for the classification of monoclonal gammopathies, multiple myeloma and related disorders: a report of the International Myeloma Working Group. Br J Haematol 2003;121(5):749–757.

2. Attal M, Lauwers-Cances V, Hulin C, et al. Lenalidomide, bortezomib, and dexamethasone with transplantation for myeloma. N Engl J Med 2017;376(14):1311–1320.

3. Traube FR, Schiffers S, Iwan K, et al. Isotope-dilution mass spectrometry for exact quantification of noncanonical DNA nucleosides. Nat Protoc 2019;14(1):283–312.

4. Martin M. Cutadapt removes adapter sequences from high-throughput sequencing reads. EMBnet.journal 2011;17(1):10.

5. Langmead B, Salzberg SL. Fast gapped-read alignment with Bowtie 2. Nat Methods 2012;9(4):357–359.

6. Ashoor H, Hérault A, Kamoun A, et al. HMCan: a method for detecting chromatin modifications in cancer samples using ChIP-seq data. Bioinformatics 2013;29(23):2979–2986.

7. Boeva V, Louis-Brennetot C, Peltier A, et al. Heterogeneity of neuroblastoma cell identity defined by transcriptional circuitries. Nat Genet 2017;49(9):1408–1413.

8. Ross-Innes CS, Stark R, Teschendorff AE, et al. Differential oestrogen receptor binding is associated with clinical outcome in breast cancer. Nature 2012;481(7381):389–393.

9. Saint-André V, Federation AJ, Lin CY, et al. Models of human core transcriptional regulatory circuitries. Genome Res 2016;26(3):385–396.

10. Agirre X, Castellano G, Pascual M, et al. Whole-epigenome analysis in multiple myeloma reveals DNA hypermethylation of B cell-specific enhancers. Genome Res 2015;25(4):478–487.

11. Subramanian A, Tamayo P, Mootha VK, et al. Gene set enrichment analysis: A knowledge-based approach for interpreting genome-wide expression profiles. Proc Natl Acad Sci 2005;102(43):15545–15550.

12. Ernst J, Kellis M. ChromHMM: automating chromatin-state discovery and characterization. Nat Methods 2012;9(3):215–216.

13. ENCODE Project Consortium. An integrated encyclopedia of DNA elements in the human genome. Nature 2012;489(7414):57–74.

14. Davis CA, Hitz BC, Sloan CA, et al. The Encyclopedia of DNA elements (ENCODE): data portal update. Nucleic Acids Res 2018;46(D1):D794–D801.

15. Cleynen A, Szalat R, Samur Kemal M, et al. Expressed fusion gene landscape and its impact in multiple myeloma. Nat Commun 2017;8(1):1–10.

16. Dobin A, Davis CA, Schlesinger F, et al. STAR: ultrafast universal RNA-seq aligner. Bioinformatics 2013;29(1):15–21.

17. Liao Y, Smyth GK, Shi W. featureCounts: an efficient general purpose program for assigning sequence reads to genomic features. Bioinformatics 2014;30(7):923–930.

18. Jin Y, Chen K, De Paepe A, et al. Active enhancer and chromatin accessibility landscapes chart the regulatory network of primary multiple myeloma. Blood 2018;131(19):2138–2150.

19. Terranova C, Tang M, Orouji E, et al. An integrated platform for genome-wide mapping of chromatin states Using High-throughput ChIP-sequencing in Tumor Tissues. J Vis Exp;(134): [Epub ahead of print].
